# Impact of a Best Practices Program in Patients with Relapsed/Refractory Multiple Myeloma Receiving Selinexor

**DOI:** 10.3390/curroncol31010034

**Published:** 2024-01-14

**Authors:** Lucio N. Gordan, David Ray, Stephen C. Ijioma, George Dranitsaris, Amanda Warner, Trevor Heritage, Matthew Fink, David Wenk, Paul Chadwick, Natasha Khrystolubova, Shachar Peles

**Affiliations:** 1 Florida Cancer Specialists and Research Institute, Tampa, FL 33609, USA; 2 Karyopharm Therapeutics Inc., Newton, MA 02459, USA; 3 Department of Public Health, Syracuse University, Syracuse, NY 13244, USA

**Keywords:** multiple myeloma, selinexor, best practices, impact

## Abstract

Best practice (BP) in cancer care consists of a multifaceted approach comprising individualized treatment plans, evidence-based medicine, the optimal use of supportive care and patient education. We investigated the impact of a BP program in patients with relapsed/refractory multiple myeloma (RRMM) receiving selinexor. Features of the BP program that were specific to selinexor were initiating selinexor at doses ≤80 mg once weekly and the upfront use of standardized antiemetics. Study endpoints included time to treatment failure (TTF), duration of therapy, dose limiting toxicities and overall survival. Comparative analysis on TTF and duration of therapy was conducted using a log-rank test and multivariate Cox proportional hazard regression. Over the ensuing 12-month post-BP period, 41 patients received selinexor-based therapy compared to 68 patients who received selinexor-based therapy pre-BP implementation. Patients treated in the post-BP period had reductions in TTF (hazard ratio [HR] = 0.50; 95% CI: 0.27 to 0.92). Patients in the pre-BP period were four times more likely to stop therapy than those in the post-period (odds ratio [OR] = 4.0, 95% CI: 1.75 to 9.3). The findings suggest a BP program tailored to selinexor could increase the time to treatment failure, increase treatment duration and lower the incidence of drug limiting toxicities.

## 1. Introduction

Multiple myeloma (MM) is a malignant neoplasm of clonal B cells originating in the bone marrow, with the median age at diagnosis being 69 years [1]. A goal of MM treatment is to prolong overall survival, avoid disease sequela and improve patient quality of life (QOL). The last two decades have been transformative for myeloma patients, with many new drugs gaining approval, including the immunomodulatory agents, proteasome inhibitors, anti-CD38 monoclonal antibodies, nuclear transporter protein exportin-1 (XPO-1) inhibitor, and chimeric antigen receptor T-cell (CAR-T) therapies [2,3]. However, despite the emergence of new therapies, most patients will relapse due to residual resistant MM clones [4,5]. To prolong duration of response and overall survival, the optimal strategy is to maximize the clinical utility of currently available agents.

The Affordable Care Act (ACA), enacted in March 2010, had a primary goal of improving value via achieving better patient health outcomes at a lower per capita cost, which included provisions to expand value-based care [6]. This built upon the Health Information Technology for Economic and Clinical Health (HITECH) Act [7], which incorporated incentive payments to increase the “meaningful use” of electronic health records (EHRs). This resulted in the widespread use of EHRs to inform individualized care, monitor health care delivery and improve patient outcomes [6,8]. These policy changes led to value-based care initiatives such as the Oncology Care Model (OCM), which incentivized practitioners to improve the way in which they provide cancer care to patients, and to avoid unnecessary costs [9]. These policy changes incentivized practices to address the complex care needs of patients receiving chemotherapy while increasing focus on best practice services that will improve patient experience or health outcomes [9]. 

Florida Cancer Specialists and Research Institute (FCS) implemented a BP program on 1 March 2022. The BP program was tailored to address the drug delivery challenges associated with individual anticancer drugs, such as selinexor. Selinexor is an oral selective nuclear export inhibitor that inhibits the nuclear transporter protein exportin 1, leading to the accumulation of tumor suppressor proteins in the nuclei of malignant cells and blocks protein translation of oncogenes that drive cell proliferation, ultimately causing cell cycle arrest and apoptosis [10]. The following four selinexor combinations are included in the National Comprehensive Cancer Network (NCCN) guidelines for patients with RRMM dependent on their prior treatment: selinexor–bortezomib–dexamethasone (XVd), selinexor–carfilzomib–dexamethasone (XKd), selinexor–pomalidomide–dexamethasone (XPd), and selinexor–daratumumab–dexamethasone (XDd) in patients with relapsed/refractory MM (RRMM)^+^ [11].

The BP program implemented at FCS aimed to keep patients adherent and compliant with their therapy, with the goal being to optimize clinical outcomes. It involved educating providers on selinexor dosing guidelines and toxicity management in RRMM. As part of the patient management workflow, the clinical team developed drug-specific protocols detailing the entire management process from initiation of therapy to discontinuation. For each new selinexor-based regimen, the clinical pharmacist submitted a request to the treating physician to also prescribe antiemetic therapies such as ondansetron, olanzapine and/or rolapitant prescriptions if none had been ordered. This allowed the practice to undertake a proactive approach in addressing the more common selinexor adverse events (AEs) such as nausea and vomiting. In addition, the counseling pharmacist scheduled a follow-up call with the patient to monitor drug intolerance, reinforce the importance of antiemetic therapy, and reach out to the prescriber if further interventions were needed. The BP program also initiated selinexor at doses ≤80 mg once weekly. Specifically, the EHR system was pre-populated with a lower selinexor starting dose of 80 mg or less. FCS selected the 80 mg selinexor dose threshold as it is the first dose-reduction step recommended in the package insert. This prompted physicians to start with a lower selinexor starting dose, with an option to change the starting dose as deemed fit. Prior to this EHR update, selinexor was either pre-populated with a higher starting dose or left blank for the provider to select the starting dose. The intent of the BP program was to prolong the duration of clinical benefit from selinexor and to reduce dose limiting toxicities (DLTs). The current study investigated the impact of the BP program in patients with RRMM receiving selinexor. 

^+^As of publication, XVd is the only regimen approved by the U.S. FDA in RRMM.

## 2. Materials and Methods

This was a retrospective, observational study using EMR data from FCS to evaluate outcomes in RRMM patients treated with a selinexor-based regimen pre- and post-implementation of the BP program. The study utilized a pre vs. post design where the post-period was defined as the 12-month time frame following the implementation of the BP program. For inclusion into this study, patients were required to be 18 years of age or older, diagnosed with RRMM, and received a selinexor-based regimen as part of routine clinical care. Patients were excluded if they were enrolled in any clinical trial during selinexor treatment. Patients in the pre-implementation period (cohort 1) consisted of those who started a selinexor-based regimen before 1 March 2022 (date of BP implementation) while the post-implementation period (cohort 2) consisted of those who started a selinexor-based regimen between 1 March 2022 and 1 March 2023. The study endpoints were time to treatment failure (TTF), duration of therapy, the frequency of DLTs and overall survival. TTF was defined as the time from the start of selinexor to disease progression, discontinuation because of drug toxicity or death.

### 2.1. Data Collection

Prior to the start of treatment, data collection consisted of patient demographics, disease characteristics, Eastern Cooperative Oncology Group (ECOG) performance status, median duration of disease, disease stage and cytogenetics and all prior therapies. Data collection specific to selinexor included concomitant drugs administered, line of therapy, dose at the start and completion of therapy as well as all dose modifications, delays, schedule changes and discontinuations (DCs). Drug DCs were further assessed and the reason(s) for terminating therapy was collected, including the contributing toxicity in cases where an AE was the underlying cause. All the information was collected via a standardized electronic data collection form. The study protocol was reviewed and approved by the FCS Ethics Review Board and Western Institutional Review Board.

### 2.2. Sample Size and Statistical Considerations

The current study was deemed exploratory, and a formal sample size estimate was not determined. Demographic data, disease characteristics, current and prior RRMM therapies, AEs and all clinical outcomes’ data were presented as descriptive statistics as means, medians or proportions with appropriate measures of variance, such as 95% CI and interquartile range (IQR). The comparative analysis on TTF and duration of therapy was conducted using a log-rank test and multivariate Cox proportional hazard regression. The TTF survival curve was generated through the Kaplan–Meier estimator method, using a censoring date of 1 March 2023. The likelihood ratio test was used in a backwards elimination process (*p* < 0.05 to retain) to retain the final variables for inclusion into the Cox multivariate model. The pre- vs. post-implementation period was the primary independent variable and was retained in the model, notwithstanding. The frequency of all-cause treatment DCs and DCs due to AEs were compared using the Fisher’s exact test. There were no statistical adjustments for multiple comparisons.

A concern in real-world evidence studies is bias from unmeasured confounding, that is, some third variable related to both the intervention and outcome that might explain the association [12]. E-values measure the strength of the correlation that an unmeasured confounder would need to have with both the intervention and the outcome variable to fully explain away a specific treatment–outcome association [13]. In the current study, E-values were calculated from the hazard ratio (HR) for TTF derived from the pre- vs. post-implementation comparison.

## 3. Results

Data were collected on a total of 109 patients; of these, 68 patients received selinexor during the pre-implementation period and were compared to 41 patients prescribed selinexor in the post-period. Patients in both periods were similar in age, gender, body mass index (BMI), performance status, type of MM and prior exposure to lenalidomide, pomalidomide, bortezomib and daratumumab (Table 1). There were, however, more stage III patients in the pre-implementation period than in the post-period (38.2% vs. 31.7%). Furthermore, more patients in the pre-implementation period had prior exposure to carfilzomib (91.2% vs. 70.7%) and isatuximab (11.8% vs. 7.3%). The median follow-up time for disease progression or death was 24.0 (13.8–41.6) and 6.7 (1.3–11.3) months in the pre- and post-cohorts, respectively.

Selinexor was primarily used by patients in the fifth or greater line of therapy (pre-period = 86.8%; post-period = 85.4%). The use of selinexor as a doublet therapy declined from 42.7% to 14.6% in the pre- and post-implementation period, respectively, with a concurrent increase in the utilization of triplet therapy from 54.5% to 85.5%. More patients initiated selinexor at doses ≤80 mg once weekly in the post-implementation period compared to the pre-period (78.0% vs. 48.5%) (Table 2). Schedule changes and treatment discontinuations were more common in the pre-implementation period (Table 2). Patients in the pre-period were approximately four times more likely to stop therapy than those in the post-period (OR = 4.0, 95% CI: 1.75 to 9.3). The most common reasons for treatment DCs were disease progression and AEs, both of which were numerically higher in patients treated during the pre-implementation period (Table 2). The median duration of therapy was 2.5 (1.2 to 4.4) and 4.4 months (IQR: 1.1 to 9.4) during the pre- and post-implementation periods, respectively.

**Figure 1:**
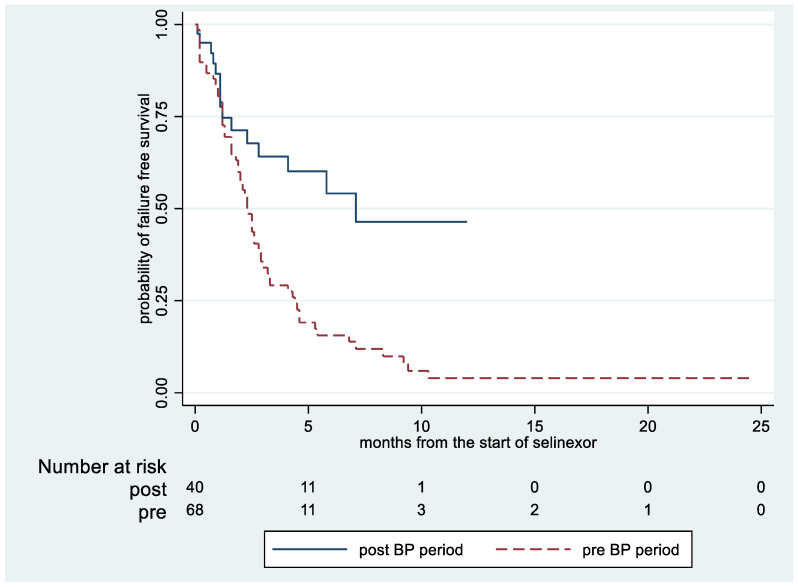
Time to treatment failure during the pre- and post-best practice implementation period.

DCs due to AEs occurred in 44.1% of patients in the pre-implementation period compared to 19.5% in the post-period (OR = 2.9, 95%CI: 1.18 to 7.2). Some of the AEs leading to treatment DCs (pre- vs. post-implementation period) were nausea (22.1% vs. 9.8%), vomiting (8.8% vs. 2.4%), fatigue (17.7% vs. 7.3%) and thrombocytopenia (13.2% vs. 4.9%). AEs leading to DCs that were similar between groups included diarrhea, constipation, anemia and neutropenia (Table 3). As of the censoring date of 1 March 2023, 14 of 41 (34.2%) of patients in the post-period remained on therapy compared to none in the pre-period. The most common cause of death was disease-related based on physician documentation (Table 4). The median TTF was 2.3 months (IQR: 1.2 to 4.4) in the pre-period vs. 7.1 months (IQR: 1.2 to NR) in the post-period (Figure 1).

The parameters pre- vs. post-implementation period, dose modifications, changes in the dosing schedule and the selinexor starting dose were strongly associated with TTF. Patients receiving selinexor following the implementation of the BP program had a 50% reduction in the risk of treatment failure compared to similar patients who initiated therapy before the start of the program (HR = 0.50; 95% CI: 0.27 to 0.92). Furthermore, dose modifications or selinexor scheduling changes had a positive impact on delaying TTF (Table 5). Using a dose of ≤60 mg as the reference, patients who started selinexor treatment at the 100 mg or ≥120 mg doses were more likely to experience a treatment failure (Table 5). Patients receiving higher doses of 100 mg or ≥120 mg doses were 2.5 and 5.4 times, respectively, more likely to fail therapy than those who started at a dose of ≤60 mg (Table 5). There were no statistically significant differences in the risk of treatment failure between patients who started selinexor at the 80 mg dose when compared to those who started on the ≤60 mg dose. E-values were then used to assess unmeasured confounding of TTF. E-values with 95%CIs were generated from the HR for TTF (0.50) using the approach described by VanderWeele and Ding, 2017 [13]. The E-value for the HR of TTF was estimated to be 3.25 (95%CI: 1.21–6.60). 

The proportion of patients alive at six months following the start of selinexor was projected to be 57.0% (44.3% to 67.8%) and 73.6% (55.1% to 85.4%) in the pre- and post-period, respectively. At 12 months following the start of selinexor, the overall survival was projected to be 38.2% (26.6% to 50.0%) in the pre-period and 51.6% (24.8% to 73.0%) in the post-period (Table 4).

## 4. Discussion

This retrospective observational study using a pre vs. post design was conducted to assess the impact of a BP program on the delivery of selinexor in patients with RRMM. Aspects of the program that were customized to selinexor included a recommended starting dose of ≤80 mg once weekly and standardized antiemetics consisting of ondansetron, rolapitant and/or olanzapine. The findings of the investigation revealed that patients who received selinexor under a BP program remained on therapy longer, had a significant increase in TTF, a lower frequency of DLTs and fewer treatment DCs due to drug toxicity or disease progression. Following the implementation of the BP program, the selinexor duration of treatment doubled while DCs due to AEs halved. The longer time to treatment failure observed in the post-implementation period could be attributed to the decrease in selinexor doublet therapy and the increase in triplet therapy from the pre- to post-implementation period. 

The real-world findings suggesting improved time to treatment failure with lower dose and triplet therapy from this program have been observed in the selinexor clinical trial setting. Post-hoc and subgroup analyses of the BOSTON randomized trial showed that patients who underwent selinexor dose reductions had improved efficacy (median progression-free survival: 16.6 months vs. 9.2 months), reduced AE rates and suggest an improved QOL compared to patients without dose reductions. The starting dose of selinexor on that trial was 100 mg weekly with prespecified dose reductions for AEs being 80 mg weekly, 60 mg weekly and 40 mg weekly [14,15]. Nausea, vomiting, fatigue and thrombocytopenia were the treatment toxicities whose incidence and severity were most impacted in the post-period under the BP program. NCCN guidelines on antiemesis, emerging data, the selinexor package insert and clinical practice experience recommend administration of 5-HT3 receptor antagonists (e.g., ondansetron) or neurokinin-1 (NK1) receptor antagonists (e.g., aprepitant and rolapitant) and/or low-dose olanzapine both before and following selinexor administration [16]. Various initiatives such as the HITECH Act led to the widespread use of EHRs to inform personal care and monitor health service delivery and patient outcomes [6]. By implementing a BP program via EHRs, physicians were better educated on the effective use of selinexor, and the initiation of supportive therapy, which resulted in improved clinical outcomes. The findings from this study support the hypothesis that a lower starting dose of selinexor, along with evidence-based primary antiemetic prophylaxis, can improve patient clinical outcomes, and reduce the incidence of severe DLTs. Additional benefits that may have been derived include improvements in patient QOL and reductions in health care costs secondary to a lower frequency of drug toxicities and less need for additional medical care.

This study has several limitations to be acknowledged. This was a retrospective observational investigation using a pre vs. post design and not a randomized trial. Such designs are at risk for temporal bias, where changes observed between the before and after phases may be influenced by external events or factors unrelated to the intervention. There is also a risk of selection bias because the sample of subjects included in the before period may have had specific unmeasured characteristics that differed from the group that was managed under the BP program. This bias can limit the generalizability of the findings and affect the validity of the present study’s results. The challenge of all real-world studies is both measured and unmeasured confounding variables. We attempted to address measured confounding by conducting a multivariate analysis on the primary endpoint, which can adjust for measured confounders. E-values were generated from the HR for TTF to assess unmeasured confounding variables. The relevant parameter in an E-value is the lower 95%CI. The interpretation is that for TTF, the low magnitude (1.21) of lower 95% CI of the E-value implies that an unmeasured confounding variable of only marginal association with the TTF could have accounted for the observed effect size. Given the retrospective nature of the data, it was also difficult to assess and quantify the severity of AEs using the established grading scales. The median follow-up time in patients treated during the pre-period was considerably longer than those in the post-period. Therefore, the overall survival results should be interpreted with caution. Patients who had their selinexor dose reduced likely had other clinical benefits, thus, potentially understating the effect. Despite these limitations, a BP program designed around the specific characteristics of a cancer therapy can have positive benefits in patient outcomes, reduce drug toxicity and potentially contribute to a decrease in health care resource use.

## 5. Conclusions

The implementation of a BP program tailored to selinexor where patients received lower starting doses than those approved by the FDA and in combination with standardized antiemetic therapy reduced the likelihood of treatment failure, increased treatment duration and lowered the incidence of DLTs. These findings support the hypothesis that a BP program designed around specific anticancer drugs can optimize prescribing practices, leading to better disease control and improvements in a patient’s cancer care journey.

## Figures and Tables

**Table 1:** Demographic and clinical characteristics of patients prior to the start of selinexor during the pre- and post-best practice implementation period.

Parameter	Pre-Implementation (*n* = 68)	Post-Implementation (*n* = 41)
Median age at MM diagnosis (range)	64.0 (33–80)	67.0 (40–80)
Median age at the start of selinexor (range)	69.5 (37–85)	71 (45–85)
Female sex	55.9% (38)	56.1% (23)
Median BMI (range)	26.9 (18.2–44.7)	25.8 (20.6–39.7)
Race		
White	64.7% (44)	65.0% (26)
Black	22.1% (15)	7.5% (3)
Other	13.2% (9)	27.5% (11)
Not documented	0.0% (0)	1.4% (1)
ISS at MM diagnosis		
Stage I	20.6% (14)	29.3% (12)
Stage II	25.0% (17)	14.6% (6)
Stage III	38.2% (26)	31.7% (13)
Not documented	16.2% (11)	24.4% (10)
Type of myeloma at Dx		
Active	67.7% (46)	68.3% (28)
Light chain	29.4% (20)	29.3% (12)
Other	2.9% (2)	2.4% (1)
ECOG Performance Status		
0 or 1	79.4% (54)	75.6% (31)
≥2	14.7% (10)	14.6% (6)
Not documented	5.9% (4)	9.8% (4)
Median time from Dx to the start of selinexor (years; range)	5.5 (1.5–23.1)	5.3 (1–21.5)
Cytogenetics		
t (4;14)	8.8% (6)	9.8% (4)
t (14;16)	2.9% (2)	0.0% (0)
del (17p)	19.1% (13)	9.8% (4)
gain/amp [1q21]	30.9% (21)	26.8% (11)
Prior drug exposure		
Lenalidomide	100% (68)	100% (41)
Pomalidomide	95.6% (65)	95.1% (39)
Bortezomib	97.1% (66)	97.6% (40)
Carfilzomib	91.2% (62)	70.7% (29)
Daratumumab	97.1% (66)	97.6% (40)
Isatuximab	11.8% (8)	7.3% (3)

Abbreviations: MM = multiple myeloma; ECOG: Eastern Oncology Cooperative Group; BMI = body mass index; ISS = international staging system; Dx = diagnosis.

**Table 2:** Characteristics of selinexor therapy during the pre- and post-best practice implementation period.

Parameter	Pre-Implementation (*n* = 68)	Post-Implementation (*n* = 41)
Selinexor regimen		
X	1.5% (1)	0.0% (0)
Xd	42.7% (29)	14.6% (6)
XDd	1.5% (1)	4.9% (2)
XKd	10.3% (7)	22.0% (9)
XPd	11.8% (8)	9.8% (4)
XPd + Isatuximab-irfc	1.5% (1)	0.0% (0)
XVd	30.9% (21)	48.8% (20)
Line of therapy		
Third	2.9% (2)	4.9% (2)
Fourth	10.3% (7)	9.8% (4)
≥Fifth	86.8% (59)	85.4% (35)
Selinexor starting dose		
≤60 mg	17.7% (12)	14.6% (6)
80 mg	30.9% (21)	63.4% (26)
100 mg	25.0% (17)	9.8% (4)
≥120 mg	26.5% (18)	12.2% (5)
Selinexor dose at discontinuation		
40 mg	14.7% (10)	17.1% (7)
50 mg	1.5% (1)	0.0% (0)
60 mg	20.6% (14)	31.7% (13)
80 mg	30.9% (21)	39.0% (16)
100 mg	20.6% (14)	4.9% (2)
120 mg	2.9% (2)	0.0% (0)
160 mg	8.8% (6)	7.3% (3)
Dose modifications	44.1% (30)	43.9% (18)
Dosing schedule change	17.7% (12)	14.6% (6)
Dose delays	16.2% (11)	19.5% (8)
Treatment interruptions	36.8% (25)	48.8% (20)
Other treatment modifications	2.9% (2)	2.4% (1)
Treatment discontinuations	66.2% (45)	29.3% (12)
Reason for discontinuation ^1^		
Disease progression	45.6% (31)	12.2% (5)
Adverse events	44.1% (30)	19.5% (8)
Enrollment into clinical trial	1.5% (1)	0.0% (0)
Death	5.9% (4)	7.3% (3)
Other	23.5% (16)	36.6% (15)
Lost to follow up	11.8% (8)	4.9% (2)
Still on therapy	0.0% (0)	34.2% (14)
Median duration of therapy in months (IQR) ^2^	2.5 (1.2–4.4)	4.4 (1.1–9.4)

Abbreviations: X = selinexor; D = daratumumab; d = dexamethasone; K = carfilzomib; P = pomalidomide; V = bortezomib, IQR = interquartile range. ^1^ In some patients, there were concomitant reasons that led to treatment discontinuations. ^2^ *p* = 0.037, as determined by the Log-rank test.

**Table 3:** Treatment limiting toxicities during selinexor therapy.

Parameter	Pre-Implementation (*n* = 68)	Post-Implementation (*n* = 41)
Drug discontinuation due to AEs	44.1% (30)	19.5% (8)
AEs contributing to discontinuation ^1^		
Nausea	22.1% (15)	9.8% (4)
Vomiting	8.8% (6)	2.4% (1)
Weight loss	5.9% (4)	7.3% (3)
Diarrhea	4.4% (3)	9.8% (4)
Constipation	1.5% (1)	2.4% (1)
Fatigue	17.7% (12)	7.3% (3)
Decreased appetite	11.8% (8)	9.8% (4)
Dyspnea	1.5% (1)	2.4% (1)
Asthenia	7.4% (5)	4.9% (2)
Insomnia	1.5% (1)	2.4% (1)
Dizziness	4.4% (3)	2.4% (1)
Thrombocytopenia	13.2% (9)	4.9% (2)
Anemia	1.5% (1)	2.4% (1)
Neutropenia	2.9% (2)	2.4% (1)
Leukopenia	1.5% (1)	2.4% (1)
Pneumonia	1.5% (1)	0.0% (0)
Other	5.9% (4)	4.9% (2)

Abbreviations: AEs = adverse events. ^1^ In some patients, there were concomitant AEs that led to treatment discontinuations.

**Table 4:** Patient status as of the censoring date.

Parameter	Pre-Implementation (*n* = 68)	Post-Implementation (*n* = 41)
Median follow-up (IQR), months	24.0 (13.8–41.6)	6.7 (1.3–11.3)
Treatment status		
Still on therapy	0.0% (0)	34.2% (14)
No longer on therapy	88.2% (60)	61.0% (25)
Loss to follow up	11.8% (8)	4.9% (2)
Treatment failure ^1^	86.8% (59)	36.6% (15)
Median TTF in months (IQR) ^2,3^	2.3 (1.2–4.4)	7.1 (1.2-NR)
Survival status		
Alive	13.2% (9)	65.9% (27)
Dead	75.0% (51)	29.3% (12)
Loss to follow up	11.8% (8)	4.9% (2)
Cause of death		
Disease-related ^4^	51.5% (35)	22.0% (9)
Not disease-related	11.8% (8)	0.0% (0)
Not documented	36.8% (25)	78.1% (32)
Patients alive at 6 months from the start of Selinexor ^5^ (95%CI)	57.0% (44.3–67.8%)	73.6% (55.1–85.4%)
Patients alive at 12 months from the start of Selinexor ^5^ (95%CI)	38.2% (26.6–50.0%)	51.6% (24.8–73.0%)

Abbreviations: TTF = time to treatment failure; NR = not reached. ^1^ Treatment failure was defined as disease progression, discontinuation because of drug toxicity or death. ^2^ *p* = 0.001, as determined by the Log-rank test. ^3^ The censoring date was 1 March 2023. ^4^ Disease-related defined as any death specifically related to MM based on physician documentation.^5^ Estimated using the Kaplan–Meier estimator method.

**Table 5:** Multivariate Cox regression analysis on time to treatment failure.

Variable ^1^	Hazard Ratio ^2^	(95% CI)
Post- vs. pre-best practice implementation	0.50	(0.27–0.92)
Dose modification	0.44	(0.25–0.77)
Dosing schedule change	0.26	(0.10–0.64)
Selinexor start dose (ref is ≤60 mg)		
80 mg dose	1.41	(0.66–3.00)
100 mg dose	2.52	(1.12–5.65)
≥120 mg dose	5.43	(2.25–13.08)

^1^ These are the final variables that were retained following the application of the likelihood ratio test (*p* < 0.05 to retain) in a backwards elimination process. The best practice implementation variable was the primary independent variable and was kept in the model notwithstanding. ^2^ An HR of less than one indicates a lower risk and greater than one indicates an increased risk of treatment failure.

## Data Availability

Any data sharing requests should be made to the Florida Cancer Specialists and Research Institute, Tampa, FL 33609, USA.
